# Association of the along‑perivascular space (ALPS) index and white matter volume with cognitive dysfunction in relapsing‑remitting multiple sclerosis patients

**DOI:** 10.1186/s12883-026-04826-4

**Published:** 2026-03-27

**Authors:** Yuanjun Song, He Zhao, Qiong Wu, Shenghui Xie, Jinlong He, Rong Bai, Shaoyu Wang, Yang Gao

**Affiliations:** 1 Inner Mongolia Medical University, Hohhot, Inner Mongolia China; 2 Department of Radiology, Affiliated Hospital of Inner Mongolia Medical University, Hohhot, Inner Mongolia China; 3 Magnetic Resonance Research Collaboration Department, Siemens Healthineers, Shanghai, China

**Keywords:** RRMS, Relapsing remitting multiple sclerosis, ALPS, Along perivascular space, NWMV, Normalized white matter volume

## Abstract

**Objective:**

To investigate the relationship among the along perivascular space (ALPS) index, conventional neuroimaging markers, and cognitive impairment in patients with relapsing–remitting multiple sclerosis (RRMS).

**Methods:**

This study included 45 RRMS patients and 41 healthy controls (HC). All participants underwent clinical evaluation, magnetic resonance imaging (MRI), and comprehensive cognitive assessments. Cognitive function was assessed using the Brief International Cognitive Assessment for MS (BICAMS), which includes the Symbol Digit Modalities Test (SDMT), California Verbal Learning Test–II (CVLT-II), and Brief Visuospatial Memory Test–Revised (BVMT-R). Additional tests evaluated attention, executive function, verbal fluency, and global cognition. Cognitive impairment was defined as a Z‑score ≤ -1.5 in at least two of the three BICAMS domains. Spearman correlation and multiple linear regression analyses were used to examine associations between neuroimaging metrics and cognitive scores in RRMS patients. Backward stepwise logistic regression was performed to identify variables independently associated with cognitive impairment.

**Results:**

RRMS patients had a significantly lower ALPS index compared to HC (t = -2.225, q = 0.029). Within the RRMS group, patients with cognitive impairment showed further reductions in the ALPS index. Multiple linear regression confirmed that the ALPS index was significantly associated with Z‑scores of the Symbol Digit Modalities Test (SDMT; β = 0.415, q = 0.003), PASAT-3 s (Paced Auditory Serial Addition Test-3 s; β = 0.305, q = 0.037), PASAT-2 s (β = 0.429, q = 0.002) and Controlled Oral Word Association Test (COWA; β = 0.305, q = 0.044). In addition, a negative correlation was observed between white matter lesion number (WMLN) and the Z‑scores of COWA (β = -0.340, q = 0.013). In the backward stepwise logistic regression model, the ALPS index (OR = 0.044, 95% CI: 0.003–0.776, q = 0.033) and normalized white matter volume (nWMV; OR = 0.764, 95% CI: 0.599–0.975, q = 0.030) were retained as variables independently associated with cognitive impairment.

**Conclusion:**

Our study suggests that both a reduced ALPS index and lower nWMV are independently associated with cognitive impairment in RRMS. These findings implicate the combined roles of impaired interstitial fluid clearance and structural integrity in RRMS-related cognitive dysfunction. As a non-invasive MR metric, the ALPS index may serve as a potential complementary biomarker for assessing cognitive risk in RRMS.

## Introduction

Multiple sclerosis (MS) is an immune-mediated disease of the central nervous system characterized by focal inflammatory demyelination, axonal injury, and progressive neurological dysfunction [1]. Relapsing–remitting multiple sclerosis (RRMS) is the most common clinical phenotype, predominantly affecting young and middle-aged adults. Besides motor, sensory, and visual impairments, cognitive dysfunction is a significant but often under-recognized clinical manifestation [2]. Studies have shown that some RRMS patients experience cognitive deficits such as slowed information processing speed, memory decline, and executive dysfunction early in the disease course, which substantially impact occupational and social functioning [3].

Although previous research has demonstrated that brain atrophy, white matter lesions, and microstructural damage have been demonstrated to be associated with cognitive impairment in patients with RRMS [4, 5], the strength of these associations is inconsistent across studies. Consequently, there is a need to integrate more advanced, multidimensional imaging biomarkers and explore alternative pathophysiological mechanisms [6].Taoka et al. [7] proposed the along the perivascular space (ALPS) index in 2017 as an indirect imaging marker of glymphatic function. Its fundamental principle is to assess the anisotropy of water molecule diffusion within the perivascular spaces surrounding deep medullary veins. Theoretically, when glymphatic function is active, increased drainage through the perivascular spaces results in enhanced diffusion along the vascular direction (x- and y-axes) relative to the perpendicular direction (z-axis). The ALPS index has been applied in the field of MS, and a lower ALPS index is associated with greater overall disability and more severe fatigue symptoms [8, 9].

In patients with RRMS, a decrease in ALPS index may lead to the accumulation of neurotoxic substances, exacerbation of neuroinflammatory reactions, and impaired synaptic function, ultimately disrupting the normal activity of cognitive networks [10]. Existing research [11, 12] suggests that the ALPS index is associated with cognitive impairment in various neurodegenerative diseases. In MS, white matter lesions and inflammatory microenvironment may further impair the structure and function of the glymphatic pathway [13]. We speculate that the ALPS index is most closely related to cognitive functions that depend on global network efficiency, such as information processing speed, attention, working memory, and episodic memory, as these cognitive domains are sensitive to metabolic clearance efficiency and neural transmission integrity.

Based on the proposed link between glymphatic dysfunction and cognitive impairment, this study was designed to test the hypothesis that the ALPS index is independently associated with cognitive deficits in RRMS, and to determine which specific cognitive domains are most closely related to this biomarker. By integrating the ALPS index with conventional structural measures, we seek to elucidate its potential role as a novel imaging marker in understanding and assessing RRMS-related cognitive decline.

## Materials and methods

### Ethics committee approval

Approval was received from the local ethical standards committee on human experimentation, and written informed consent was obtained from all subjects prior to study participation. It should be clarified that all procedures complied with the ethical standards outlined in the Declaration of Helsinki.

### Participants

This study enrolled a total of 86 participants, including 45 patients diagnosed with relapsing–remitting multiple sclerosis (RRMS group) and 41 age-, sex-, and education level-matched healthy controls (HC). All participants were recruited from the Affiliated Hospital of Inner Mongolia Medical University between March 2023 and January 2025. The inclusion criteria for the RRMS group were as follows: (1) diagnosis according to the 2017 revised McDonald criteria [14]; (2) no corticosteroid treatment within one month prior to the MRI scan, and either on long-term disease-modifying therapy (DMT) [15] or in a untreated stage. Long-term MDT was defined as the regular use of approved multiple sclerosis disease-modifying drugs for a minimum of six consecutive months, with a stable treatment regimen (no change in medication or dosage) for at least three months prior to enrollment.

Exclusion criteria for all patients and controls were as follows: (1) a history of other significant diseases, including major cardiovascular or cerebrovascular diseases that could potentially affect glymphatic function; (2) any concurrent neurological disorders (other than MS for the patient group) or significant psychiatric conditions that could impact cognition; (3) contraindications to MRI; (4) severe uncorrected visual or auditory impairments that would preclude valid cognitive testing; (5) presence of structural brain abnormalities on MRI unrelated to MS; (6) use of corticosteroids or immunomodulatory drugs within the past month.

### Cognitive function assessment

All RRMS patients underwent cognitive examination on the same day as the MRI acquisition. All tests were administered by neurologists following standardized protocols to ensure the accuracy and comparability of the results. The primary evaluation was based on the Brief International Cognitive Assessment for MS (BICAMS) [16, 17], which comprises the Symbol Digit Modalities Test (SDMT) to evaluate information processing speed, the first five recall trials of the California Verbal Learning Test–Second Edition (CVLT-II) to assess verbal memory, and the first three recall trials of the Brief Visuospatial Memory Test–Revised (BVMT-R) to evaluate visuospatial memory. By referencing normative data based on HC, RRMS patients’ raw test scores were converted into Z-scores adjusted for age, sex, and education level [18]. In line with commonly used criteria in previous MS cognitive studies, cognitive impairment was defined as having a Z-score ≤ −1.5 in at least two of the three BICAMS core cognitive domains (information processing speed, verbal memory, and visuospatial memory). The remaining patients were classified as cognitively preserved.

To further expand the analysis of specific cognitive domains, we also incorporated the Paced Auditory Serial Addition Test (PASAT-3 s and PASAT-2 s) [19] to assess attention and working memory, the Stroop Color and Word Test (SCWT) [20], which includes word reading (StroopW), color naming (StroopC), and color-word naming (StroopD) subtests, to evaluate executive control ability, and the Controlled Oral Word Association Test (COWA) [21] to assess verbal fluency. The Montreal Cognitive Assessment (MoCA) [22] and the Mini-Mental State Examination (MMSE) [23–25] were also administered. All raw scores from cognitive tests were converted to Z‑scores based on normative data derived from the HC. Neurological disability was evaluated using the Expanded Disability Status Scale (EDSS) [26].

The cognitive tests were administered in an order that graduated from less to more demanding, as follows: EDSS, MoCA/MMSE, SDMT, CVLT-II, BVMT-R, PASAT, SCWT, COWA. Brief breaks were provided between tests to minimize fatigue. All participants completed the entire test battery, with no missing cognitive data.

### MRI data acquisition

Imaging was performed using a Siemens MAGNETOM Skyra 3.0 T MRI scanner, equipped with a fixed 32-channel head and neck coil. Patients were positioned in the supine position, with the head secured using a foam pad. They were instructed to breathe calmly and evenly while keeping their head and body as still as possible to minimize motion artifacts. The scanning sequences and parameters used were as follows: Diffusion Tensor Imaging (DTI): two b-values (0 and 1000 s/mm^2^) and acquired along 64 non-collinear directions, TR 8000 ms, TE 79 ms, slice thickness 2.0 mm, FOV 256 mm × 256 mm, matrix 100 × 100, voxel size 2.6 mm × 2.6 mm × 2.0 mm, scan time 9 min 29 s. 3D Fluid-Attenuated Inversion Recovery (3D FLAIR): TR 5000 ms, TE 387 ms, slice thickness 0.9 mm, FOV 230 mm × 230 mm, matrix 256 × 256, voxel size 0.9 mm × 0.9 mm × 0.9 mm, scan time 7 min 7 s.3D T1-weighted Magnetization Prepared Rapid Gradient Echo (3D T1 MPRAGE): TR 2300 ms, TE 2.32 ms, slice thickness 0.9 mm, FOV 240 mm × 240 mm, matrix 256 × 256, voxel size 0.9 mm × 0.9 mm × 0.9 mm, scan time 5 min 21 s.

### DTI data preprocessing and alps index calculation

All images were processed using an image analysis tool named uAI Research Portal (United Imaging Intelligence) [27]. The preprocessing steps included skull stripping, T1 weighted image is first rigidly registered with DTI data to provide anatomical reference. Subsequently, DTI images were processed using MRtrix3 [28], which involved denoising, correction of Gibbs-ringing artifacts, eddy current correction, and N4 bias field correction. Diffusivity tensors were then estimated, and fractional anisotropy (FA) maps were generated and color-encoded to represent the primary diffusion directions.

**Fig. 1:**
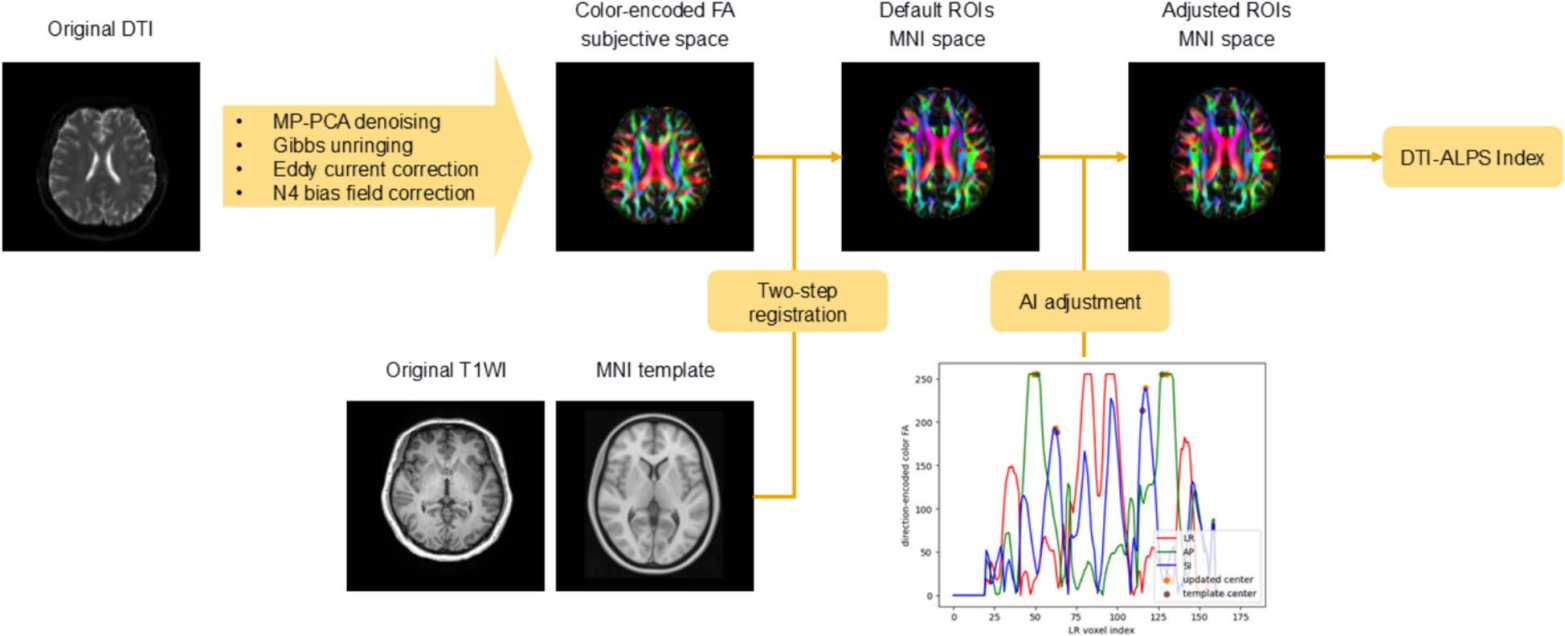
Workflow of DTI processing and ALPS index calculation

To compute the DTI ALPS-index, four circular regions of interest (ROIs) were placed within the axial plane at the intersection of the corpus callosum and cerebral ventricle. These ROIs were first identified on a standard FA template in the Montreal Neurological Institute (MNI) space, based on the methodology established by Taoka et al. [7]. The FA template was registered to the DTI space to determine the initial coordinates for the ROIs. Based on the registration results, the initial ROI position was determined. Several sets of y and z coordinates were then selected to define the cross-sectional and anteroposterior positions. For each point along the line corresponding to these coordinates, the mean tensor values in each direction were calculated within a circular region centered at that point. The x-axis corresponds to the left–right coordinate of the circle center in the image, while the red, green, and blue curves represent the mean tensor values in the left–right, anteroposterior, and superior–inferior directions, respectively. The center of the ROI was ultimately placed at the peak positions of the blue and green curves, ensuring its localization within the central regions of the projection and association fibers. An in-house algorithm was then used to automatically refine the positioning of the projection and association peaks within the color-encoded FA maps, localized to both sides of the mid-posterior corpus callosum. Spherical ROIs with a size of 3 × 3x3 mm were drawn at these locations, and the diffusion rates (Dx, Dy, Dz) in the x, y, and z directions were extracted from these regions to describe the diffusion characteristics of the projection fibers (Dxproj, Dyproj, Dzproj) and association fibers (Dxassoc, Dyassoc, Dzassoc). Finally, the ALPS index was calculated according to the following formula [29]$ALPS=\frac{mean(Dx\:\:proj, Dx\:\:assoc)}{\mathrm{m}ean(Dy\:\: proj, Dz\:\:assoc)}$. To ensure the reliability of data acquisition and reduce hemispheric dominance, we mapped ROI in both hemispheres (Fig. 1).

### Diffusion parameters based on white matter fiber skeleton

The FSL toolbox (https://fsl.fmrib.ox.ac.uk/fsl/fslwiki) was used to extract diffusion parameters based on the white matter skeleton. First, a non-linear registration was performed to map each participant’s FA image to the standard space, generating an average FA image. Secondly, The white matter fiber skeleton (FA > 0.2) was extracted, and the parameters were projected. The mean diffusivity (MD), axial diffusivity (AD), and radial diffusivity (RD) maps for each participant were projected onto the standard space using the skeleton. Finally, diffusion parameter values were extracted based on the white matter fiber skeleton.

### White matter lesion extraction and brain tissue segmentation

White matter lesions and brain tissue segmentation were performed using SPM12 software (https://www.fil.ion.ucl.ac.uk/spm/software/spm12). White matter lesion extraction was based on 3D T1-MPRAGE and 3D FLAIR images, employing the lesion growth algorithm (LGA) to obtain the white matter lesion volume (WMLV) and the white matter lesions number (WMLN).

Prior to brain tissue segmentation, white matter lesions were filled to avoid interference from low-signal lesions on structural segmentation. The filled images were visually inspected to ensure no artifacts were introduced and that the lesions were adequately filled. Subsequently, an automated method was used to segment the filled T1 images, yielding cerebrospinal fluid volume (CSFV), gray matter volume (GMV), white matter volume (WMV), total intracranial volume (TIV), and cortical thickness (CTh). To correct individual differences in head size, CSFV, WMV, and GMV were normalized by TIV, resulting in normalized cerebrospinal fluid volume (nCSFV), normalized gray matter volume (nGMV), and normalized white matter volume (nWMV).

### Statistical analysis

Statistical analyses were performed using SPSS version 26.0. The Shapiro–Wilk test was used to assess the normality of continuous variables. Variables with a normal distribution were expressed as mean ± standard deviation (± SD), and between-group comparisons were performed using independent sample t-tests. Non-normally distributed variables were expressed as median and interquartile range [M (Q1, Q3)], and comparisons were made using the Mann–Whitney U test. Categorical variables were presented as counts(n), and compared using the χ^2^ test. Spearman correlation analysis was employed to evaluate associations between the ALPS index, neuroimaging metrics, and cognitive scores in RRMS patients. Utilizing multivariate linear regression analysis to further explore the linear associations between the ALPS index, imaging indicators and various cognitive test scores, while controlling for potential confounding factors such as sex, age, and educational level.

Variables with *P* < 0.05 in the univariate analysis were selected as candidate variables for multivariate logistic regression analysis using the backward stepwise logistic regression. A binary outcome variable was established to classify RRMS patients into Co-P and Co-I groups. The discriminating ability of the model was assessed using the area under the receiver operating characteristic curve (AUROC). *P* < 0.05 indicated a statistically significant difference. To control the false positives arising from multiple comparisons, we applied false discovery rate (FDR) correction. The corrected p‑values are referred to as q‑values, and the significance threshold was set at q < 0.05.

## Results

### Demographic and clinical characteristics

A total of 45 patients with RRMS and 41 HC were included in this study. The median age of the RRMS group was 42.00 (32.5, 51.00) years, while the median age of the HC group was 46.07 (40.00, 54.00) years. The average duration of education was 10.40 years in the RRMS group and 9.94 years in the HC group. Regarding sex distribution, 31.1% of participants in the RRMS group were male and 68.9% were female, compared to 36.6% male and 63.4% female in the HC group. The median disease duration in the RRMS group was 6.40 years, and the median EDSS score was 1.68.

Based on the assessment results, a total of 45 patients with RRMS were categorized into a cognitively preserved (Co-p) group (*n* = 23) and a cognitive impairment (Co-I) group (*n* = 22). No significant difference was observed in sex distribution between the two groups. The Co-p group comprised 9 males (39.1%) and 14 females (60.9%), whereas the Co-I group comprised 5 males (22.7%) and 17 females (77.3%). However, a statistically significant difference was found in age between the two groups. The median age of patients in the Co-I group was higher than that in the Co-P group [43.00 (35.00, 60.00) years vs. 34.00 (27.00, 46.00) years; q = 0.009]. Detailed demographic and clinical information is presented in Table 1.

**Table 1:** Demographic, clinical, and MRI characteristics of the study cohort

	RRMS (*n* = 45)	HC(*n* = 41)	q-value	CO-P RRMS (*n* = 23)	CO-I RRMS (*n* = 22)	q-value
Sex (number)			0.592^a^			0.235^a^
male	14	15	-	9	5	-
female	31	26	8	14	17	-
age[years,*M*(*Q*_1_, *Q*_3_)]	42.00 (32.5, 51.00)	46.07 (40.00, 54.00)	0.083^b^	34.00 (27.00, 46.00)	43.00 (35.00, 60.00)	**0.009** ^**b**^
education[years,*M*(*Q*_1_, *Q*_3_)]	10.40 (6.5, 14)	9.94 (8, 12)	0.630^b^	12.00 (6.00, 15.00)	7.00 (6.00, 9.00)	0.927^b^
DMT patterns Regular/No (%)	37/8 (82.2, 17.8)					
Disease duration[M(Q1, Q3)]	6.40 (1.00, 10.00)	-	-	2.50 (1.00, 7.00)	8.00 (1.00,11.00)	0.139^b^
EDSS[M(Q1, Q3)]	1.68 (1.00, 2.00)	-	-	1.25(1.00, 2.25)	1.50 (0.25, 2.00)	0.566^b^
nCSFV[*M*(*Q*_1_, *Q*_3_)]	22.22 (12.60, 20.90)	18.97 (17.35, 19.65)	**0.033** ^**b**^	14.90 (12.40, 20.30)	17.70 (13.35, 24.82)	0.500^b^
nGMV($\overline{x}$±s)	45.18 ± 0.30	47.77 ± 0.60	** < 0.001** ^**c**^	48.12 ± 0.73	47.16 ± 1.12	0.459^c^
nWMV[*M*(*Q*_1_, *Q*_3_)]	35.15 (33.25, 37.60)	36.10 (34.90, 37.10)	0.534^b^	36.30 (33.17, 37.75)	32.80 (30.85, 36.70)	**0.007** ^**b**^
TIV($\overline{x}$±s)	1384.66 ± 23.89	1443.27 ± 22.81	0.081^c^	1396.17 ± 29.19	1373.37 ± 44.93	0.635^c^
CTh[mm,*M*(*Q*_1_, *Q*_3_)]	2.30 (2.23, 2.38)	2.33 (2.32, 2.40)	0.121^b^	2.32 (2.26, 2.37)	2.31 (2.22, 2.38)	0.733^b^
FA[*M*(*Q*_1_, *Q*_3_)]	0.26 (0.23, 0.29)	0.30 (0.29, 0.32)	** < 0.001** ^**b**^	0.28 (0.23, 0.29)	0.25 (0.23, 0.29)	0.376^b^
MD[*M*(*Q*_1_, *Q*_3_)]	0.49 (0.47, 0.52)	0.50 (0.49, 0.50)	0.171^b^	0.47 (0.45, 0.50)	0.50 (0.47, 0.51)	**0.022** ^**b**^
AD[*M*(*Q*_1_, *Q*_3_)]	0.74 (0.73, 0.76)	0.76 (0.76, 0.78)	** < 0.001** ^**b**^	0.73 (0.72, 0.76)	0.74 (0.73, 0.76)	0.166^b^
RD[*M*(*Q*_1_, *Q*_3_)]	0.38 (0.34, 0.41)	0.36 (0.35, 0.37)	0.318^b^	0.36 (0.32, 0.41)	0.38 (0.34, 0.40)	0.196^b^
WMLV [*M*(*Q*_1_, *Q*_3_)]	10.43 (1.72,16.57)	-	-	6.16 (1.34, 15.51)	7.45 (2.03, 15.51)	0.308^b^
WMLN($\overline{x}$±s)	18.62 ± 1.61	-	-	18.34 ± 3.05	18.90 ± 1.94	0.864^c^
ALPS index($\overline{x}$±s)	1.42 ± 0.04	1.53 ± 0.03	**0.029** ^c^	1.46 ± 0.61	1.23 ± 0.36	**0.004** ^**c**^

Bold indicates q < 0.05

*RRMS* Relapsing–remitting multiple sclerosis, *HC* Healthy controls, *Co-P* Cognitively preserved, *Co-I* Cognitively impairment, *EDSS* Expanded Disability Status Scale, *nCSFV* normalized cerebrospinal fluid volume, *nGMV* normalized gray matter volume, *nWMV* normalized white matter volume, *TIV* Total intracranial volume, *CTh* Cortical thickness, *FA* Fractional anisotropy, *MD* Mean diffusivity, *AD* Axial diffusivity, *RD* Radial diffusivity, *ALPS index* Along perivascular spaces index, *WMLV* White matter lesion volume, *WMLN* White matter lesion number

^a^Chi-square test

^b^Mann-Whitney U test

^c^Independent samples t-test

### Imaging and cognitive function parameters

As shown in Table 1, the ALPS index was significantly lower in the RRMS group compared to the HC group (*t* = −2.255, q = 0.029, Cohen ‘s d = 0.487). In terms of structural imaging parameters, RRMS patients exhibited significantly reduced nGMV (*t* = −3.726, q < 0.001, Cohen ‘s d = 0.804) and increased nCSFV (Z = 2.136, *p* = 0.033, *r* = 0.230), as well as decreased FA (Z = −5.088, q < 0.001, r = 0.549) and AD (*Z* = −4.500, q < 0.001, *r* = 0.485) compared to HC. No significant differences were observed in nWMV (*Z* = −0.623, q = 0.534, *r* = 0.067), TIV (*t* = −1.765, q = 0.081, Cohen ‘s d = 0.381), CTh (Z = −1.549, q = 0.121, r = 0.167), MD (Z = 1.370, q = 0.171, r = 0.147), and RD (Z = 0.999, q = 0.318, r = 0.107) between HC and RRMS. Compared with Co-P group, Co-I group had lower ALPS index (*t* = −3.038, q = 0.004, Cohen ‘s d = 0.656), lower nWMV (*Z* = −2.680, q = 0.007, r = 0.289), and higher MD (*Z* = 2.293, q = 0.022, r = 0.247).

As shown in Table 2, compared to HC, RRMS patients showed poorer performance across all cognitive domains assessed. Specifically, the RRMS group showed lower scores than the healthy controls on the SDMT (t = −3.167, q = 0.002), CVLT-II (Z = −4.717, q < 0.001), and BVMT-R (Z = −2.971, q = 0.003). Significant differences between RRMS and HC were also observed on the PASAT-2 s (t = −3.157, q < 0.001), PASAT-3 s (t = −2.466, q = 0.017), Stroop D (Z = 2.328, q = 0.020), Stroop W (Z = 3.031, q = 0.002), Stroop C (Z = 3.784, q < 0.001), and COWA (t = −2.594, q = 0.012). Additionally, RRMS patients scored lower than HC on the MMSE (Z = −2.492, q = 0.013) and MoCA (Z = −2.206, q = 0.027) tests.

**Table 2:** Comparison of Cognitive Test Scores between the RRMS Group and the HC Group

	cognitive domain	RRMS (*n* = 45)	HC(*n* = 41)	Statistical Values	q-value
BICAMS					
SDMT($\overline{x}$±s)	information processing speed	42.98 ± 1.94	53.96 ± 1.15	3.167^a^	**0.002**
CVLT-II[*M*(*Q*_1_, *Q*_3_)]	verbal memory	41.00 (34.00, 47.00)	59.50(51.00,62.75)	4.717^b^	** < 0.001**
BVMT-R[*M*(*Q*_1_, *Q*_3_)]	visuospatial memory	22.78(20.00,26.00)	26.19(25.00,28.00)	2.971^b^	**0.003**
Other Cognitive domain					
PASAT-2 s($\overline{x}$±s)	attention and working memory	52.42 ± 2.07	63.99 ± 1.95	3.157^a^	** < 0.001**
PASAT-3 s($\overline{x}$±s)		64.96 ± 2.16	74.63 ± 2.46	2.466^a^	**0.017**
Stroop D[*M*(*Q*_1_, *Q*_3_)]	executive control	16.00(13.00,20.50)	14.00 (12.25, 15.00)	−2.328^b^	**0.020**
Stroop W[*M*(*Q*_1_, *Q*_3_)]		25.14(16.50,31.50)	17.13(15.00,19.00)	−3.031^b^	**0.002**
Stroop C[*M*(*Q*_1_, *Q*_3_)]		40.05(26.50,47.50)	23.75(21.00,25.75)	−3.784^b^	** < 0.001**
COWA($\overline{x}$±s)	verbal fluency	39.49 ± 1.23	44.94 ± 0.63	2.594^a^	**0.012**
Overall Cognitive Assessment					
MMSE[*M*(*Q*_1_, *Q*_3_)]	-	26.84(26.00,29.00)	28.63(28.00,29.75)	2.492^b^	**0.013**
MoCA[*M*(*Q*_1_, *Q*_3_)]	-	24.51(25.25,27.00)	26.44 ± 0.33	2.206^b^	**0.027**

Bold indicates q < 0.05

*BICAMS* Brief International Cognitive Assessment for MS, *SDMT* Symbol Digit Modalities Test, *CVLT-II* California Verbal Learning Test–II, *BVMT-R* Brief Visuospatial Memory Test–Revised, *PASAT-3 s/2 s* Paced Auditory Serial Addition Test (3-s/2-s intervals), *Stroop D/W/C* Stroop Color and Word Test, *COWA* Controlled Oral Word Association Test, *MMSE* Mini-Mental State Examination, *MoCA* Montreal Cognitive Assessment

^a^Independent samples t-test

^b^Mann-Whitney U test

**Fig. 2:**
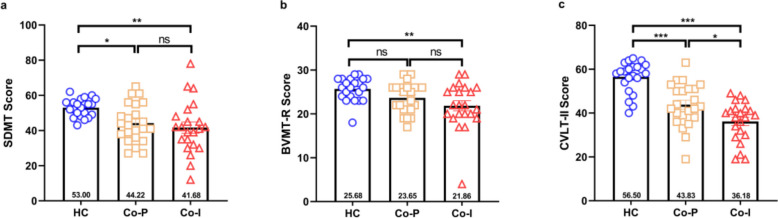
Comparison of the scores of three core scales recommended by the Brief International Cognitive Assessment for MS in co-I group, Co-P group and HC group. **a** SDMT, **b** BVMT-R, **c** CVLT-II. Co-P: cognitively preserved; Co-I: cognitive impairment; HC: healthy controls; BVMT-R, Brief Visuospatial Memory Test–Revised; SDMT, Symbol Digit Modalities Test; CVLT-II, California Verbal Learning Test–II. ***: q < 0.001, **: q < 0.01, *: q < 0.05

As shown in Fig. 2, in the SDMT scores, both Co-I (t = −3.375, q = 0.003) and Co-P (t = −2.648, q = 0030) groups scored lower than HC, while no significant difference was observed between the Co-I and Co-P groups (t = 0.764, q > 0.999). In the BVMT-R scores, the Co-I group scored lower than the HC group (t = −3.184, q = 0.006), whereas no statistically significant differences were found between the Co-P and HC groups(t = 1.711, q = 0.275), or between the Co-I and Co-P groups(t = 1.508, q = 0.409). In the CVLT-II scores, both the Co-I-(t = −7.711, q < 0.001) and Co-P (t = −4.863, q < 0.001) groups scored lower than the HC group. Furthermore, the Co-I group scored lower than the Co-P group (t = −2.933, q = 0.014).

### Correlation between cognitive performance and imaging metrics

**Fig. 3:**
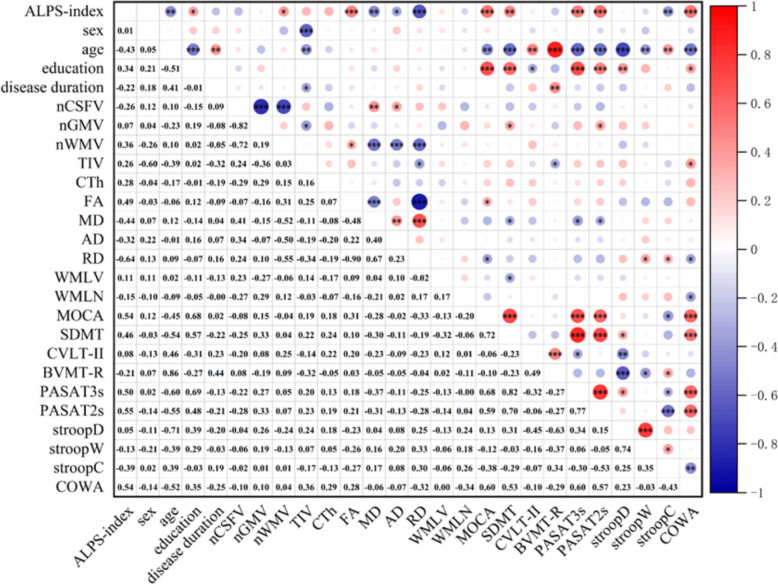
Heatmap of Correlation Analysis Between the ALPS Index, Clinical, Imaging Variables, and Cognitive Performance. ALPS index, along perivascular spaces index; nCSFV, normalized cerebrospinal fluid volume; nGMV, normalized gray matter volume; nWMV, normalized white matter volume; TIV, total intracranial volume; CTh, cortical thickness; FA, fractional anisotropy; MD, mean diffusivity; AD, axial diffusivity; RD, radial diffusivity; WMLV, white matter lesion volume; WMLN, white matter lesion number; MoCA, Montreal Cognitive Assessment; SDMT, Symbol Digit Modalities Test; CVLT-II, California Verbal Learning Test–II; BVMT-R, Brief Visuospatial Memory Test–Revised; PASAT-3 s/2 s, Paced Auditory Serial Addition Test (3-s/2-s intervals); Stroop D/W/C, The Stroop Color and Word Test; COWA, Controlled Oral Word Association Test. ***: *p* < 0.001, **: *p* < 0.01, *: *p* < 0.05

As shown in Fig. 3, among RRMS patients, the Z‑scores of SDMT, PASAT-3 s, PASAT-2 s, and COWA were positively correlated with the ALPS index (all *p* < 0.01). However, the Z‑scores of StroopC was negatively correlated with the ALPS index (*p* < 0.01). In addition, in the overall cognitive assessment, MoCA was positively correlated with the ALPS index (*p* < 0.001). Moreover, Z-scores of some cognitive tests were also correlated with certain imaging indicators (Fig. 3).

**Fig. 4:**
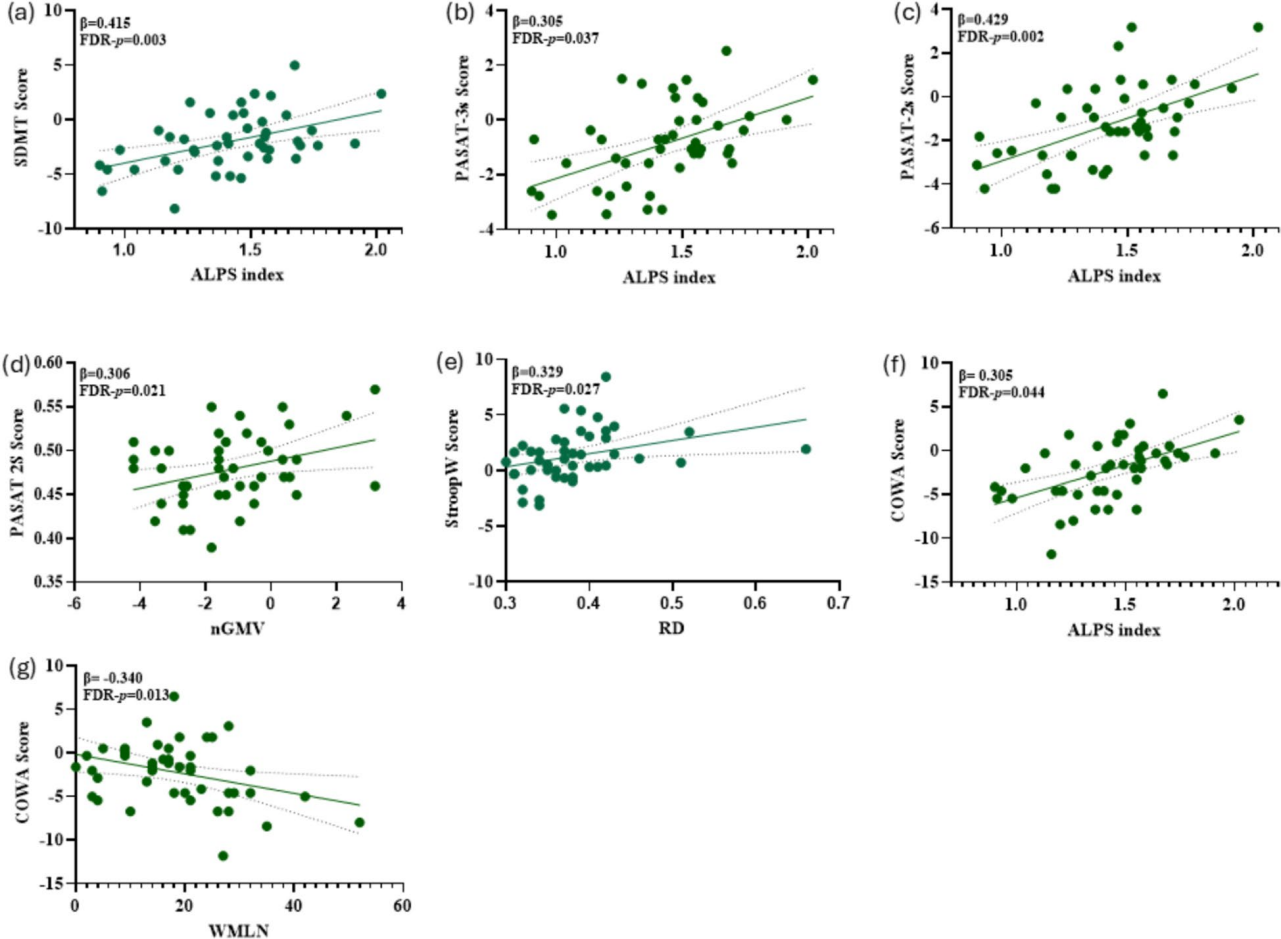
**a**-**g** Associations between ALPS index, imaging indicators, and cognitive measures in RRMS patients. Multivariate linear regression analysis was adjusted for age, sex, and educational level. SDMT, Symbol Digit Modalities Test; ALPS index, along perivascular space index; WMLN, white matter lesion number; PASAT-3 s/2 s, Paced Auditory Serial Addition Test (3-s/2-s intervals); StroopW, The Stroop Word Test; nGMV, normalized gray matter volume; RD, radial diffusivity; COWA, Controlled Oral Word Association Test. DR-p: False Discovery Rate adjusted *p*-value

Based on the results of the correlation analysis, indicators that were significantly correlated with the cognitive tests (*P* < 0.05) were selected. Further multivariate linear regression analyses were conducted for those cognitive tests. The results showed that Z‑scores of SDMT (β = 0.415, FDR-p = 0.003), PASAT-3 s (β = 0.305, q = 0.037), PASAT-2 s (β = 0.429, q = 0.002) and COWA (β = 0.305, q = 0.044) was positively correlated with the ALPS index. In addition, the Z‑scores of PASAT-2 s was positively correlated with nGMV (β = 0.306, q = 0.021). The Z‑scores of StroopW was positively correlated with RD (β = 0.329, q = 0.027). In contrast, a negative correlation was observed between the Z‑scores of COWA and WMLN (β = −0.340, q = 0.013) (Table 3 and Fig. 4).

**Table 3:** Multivariate Linear Regression Analysis of Neuropsychological Tests with Clinical and Imaging-Related Factors in RRMS Patients after adjusting for age, sex, and education level

	**B(95%CI)**	**t-value**	**q-value**	**VIF**
SDMT				
ALPS index	4.525 (1.662, 7.388)	3.195	**0.003**	1.082
nGMV	0.149(−0.02, 0.318)	1.787	0.082	1.104
MD	−5.838 (−16.522, 4.846)	−1.106	0.276	1.106
WMLV	−0.057(−0.117, 0.004)	−1.903	0.064	1.080
BVMT-R				
TIV	0.006 (0.001, 0.012)	2.203	0.133	1.000
PASAT-3 s				
ALPS index	1.928(0.121, 3.734)	2.153	**0.037**	1.081
MD	−6.611(−13.126, 0.055)	−2.002	0.052	1.081
PASAT-2 s				
ALPS index	3.287 (1.264, 5.309)	3.281	**0.002**	1.082
nGMV	0.137(0.022, 0.251)	2.407	**0.021**	1.024
MD	−4.220(−11.78, 3.329)	−1.129	0.266	1.106
Stroop W				
RD	11.520(1.346, 21.694)	2.284	**0.027**	1.000
Stroop C				
ALPS index	3.835 (−3.651, 11.320)	−1.034	0.307	1.170
RD	−20.105(−47.164, 7.153)	1.488	0.144	1.170
COWA				
ALPS index	4.541(0.121, 9.885)	2.077	**0.044**	1.309
TIV	0.004 (−0.002, 0.011)	1.432	0.160	1.251
RD	−4.234 (−20.181, 11.714)	−0.537	0.595	1.285
WMLN	−0.110 (−0.196, −0.024)	−2.589	**0.013**	1.046
MOCA				
ALPS index	0.287(−0.375, 8.597)	1.851	0.071	1.197
FA	0.150(−32.181, 52.714)	0.463	0.646	5.245
RD	−0.086(−39.196, 31.024)	−0.255	0.800	5.654

Bold indicates q < 0.05

*SDMT* Symbol Digit Modalities Test, *ALPS index* Along perivascular space index, *nGMV* normalized gray matter volume, *WMLV* White matter lesion volume, *BVMT-R* Brief Visuospatial Memory Test–Revised, *FA* Fractional anisotropy, *MD* Mean diffusivity, *PASAT-3 s* Paced Auditory Serial Addition Test-3 s interval, *PASAT-2 s* Paced Auditory Serial Addition Test-2 s interval, *Stroop W/C* The Stroop Color and Word Test, *RD* Radial diffusivity, *COWA* Controlled Oral Word Association Test, *TIV* Total intracranial volume, *WMLN* White-matter lesion number, *MoCA* Montreal Cognitive Assessment

### Independent predictors of cognitive impairment

As shown in Table 4, in the stepwise backward logistic regression model for cognitive impairment, variables such as age, sex, disease duration EDSS score, nCSFV, nGMV, TIV, FA, MD, AD, RD, WMLN and WMLV were all excluded. The final model retained only the ALPS index (OR = 0.044, 95% CI: 0.003–0.776, q = 0.033) and nWMV (OR = 0.764, 95% CI: 0.599–0.975, q = 0.030).

**Table 4:** Results of backward stepwise logistic regression analysis for discriminating factor of cognitive impairment

	**B**	**OR(95%CI)**	***P*** **-value**
ALPS index	−3.115	0.044(0.003–0.776)	0.033
nWMV	−0.269	0.764(0.599–0.975)	0.030

*ALPS index* Along perivascular space index, *nWMV* normalized white matter volume

We further conducted ROC analysis and the results showed that the AUC of the individual models of the ALPS index (AUC = 0.717) and nWMV (AUC = 0.733).

## Discussion

This study reveals that in RRMS patients, particularly those with cognitive impairment (Co-I), the ALPS index is significantly reduced. Furthermore, lower ALPS index was positively correlated with SDMT, PASAT-3 s, PASAT-2 s and COWA scores. Stepwise backward logistic regression further revealed that both a lower ALPS index and reduced nWMV were independently associated with cognitive impairment. Moreover, this result aligns with findings from studies on Alzheimer's disease and cerebral small vessel disease [11, 12], which also report a link between a decreased ALPS index and cognitive impairment. Collectively, these observations point to the possibility that the ALPS index may association with cognitive deficits.

We found that average FA and AD decreased, nGMV decreased, and nCSFV increased in RRMS patients, reflecting the multidimensional brain injury pattern in RRMS patients, including impaired axonal integrity, reduced neuronal contraction and synaptic connections, and cerebrospinal fluid retention. The ALPS index quantifies the diffusion efficiency of CSF along the perivascular spaces and has been shown to partially reflect the function of the glymphatic system [30]. We found that the ALPS index is reduced in RRMS patients. Since RRMS typically affects the deep white matter regions surrounding the ventricles, the dysfunction of clearance is particularly pronounced in these areas. This leads to impaired clearance of metabolic waste, promoting the deposition of harmful substances within the brain, triggering neuroinflammation, axonal dysfunction, and synaptic damage, thereby forming a vicious cycle of inflammation, metabolic accumulation, and neuronal injury [31]. Recent studies comparing post-mortem histopathological analysis of the brain with pre-mortem diffusion MRI data suggest that the ALPS index does not directly represent lymphatic flow. Instead, it serves as a comprehensive indicator of perivascular space diffusion, microstructural integrity, and aging processes [32].Therefore, the association we observed between the ALPS index and multiple cognitive domains may suggest that multiple coexisting and interrelated pathophysiological processes collectively impair cognitive networks. Reduced perivascular diffusion could reflect a decline in the efficiency of the glymphatic system in clearing metabolic waste products (such as β-amyloid), leading to the accumulation of neurotoxic substances and affecting neuronal function [33]. Meanwhile, damage to tissue microstructural integrity may compromise the integrity and efficiency of neural conduction. Age-related changes encompass factors such as vascular stiffening and alterations in the extracellular matrix associated with aging, serving as fundamental risk factors for cognitive decline.

Furthermore, our study identified normalized white matter volume (nWMV) as a key and independent imaging variable for distinguishing between cognitively impaired and cognitively preserved patients with RRMS. This finding is consistent with extensive prior studies [34, 35], confirming that structural brain damage, particularly the loss of white matter macrostructure, plays a foundational role in RRMS-related cognitive impairment. Unlike the dynamic physiological processes suggested by the ALPS index, nWMV more directly reflects the cumulative structural burden of the disease. The reduction in nWMV represents the macroscopic endpoint of a series of complex pathological processes in RRMS. A lower nWMV indicates widespread and largely irreversible damage to brain structural integrity, which directly constrains the neural reserve available for cognitive function.

The BICAMS [36] is a tool recently proposed by researchers for specifically evaluating the cognitive function of patients with MS. Compared with the Brief Repeatable Battery of Neuropsychological Tests (BRB-N) and the Minimal Assessment of Cognitive Function in Multiple Sclerosis (MACFIMS), it has the advantages of being less time-consuming and having lower requirements for evaluators, allowing for a rapid assessment cognitive status for MS patients [17]. In this study, we compared the BICAMS scores among the cognitively preserved group, cognitively impaired group of RRMS patients, and the HC. We found that in the cognitively preserved group, the scores of CVLT-II tests were significantly lower than those of the HC group. This may suggest that the BICAMS scale is more sensitive than the MoCA in identifying subtle cognitive changes in RRMS patients.

Our findings suggest that a lower ALPS index is associated with poorer performance across multiple specific cognitive domains. A lower ALPS index was associated with poorer performance on tests of information processing speed (SDMT) and attention (PASAT-3 s, PASAT-2 s). These tasks rely on the efficient integration of information by the deep white matter regions surrounding the ventricles and subcortical structures. Therefore, the ALPS index can sensitively reveal changes in these related cognitive functions. In contrast, the ALPS index did not show significant correlations with tasks such as the Stroop D and COWA. This may be because these tasks primarily depend on the frontal cortex and its functional connections with other cortical regions.

We found that WMLV is negatively correlated with information processing speed (SDMT), meaning that the greater white matter lesion burden, the slower the information processing speed. This is consistent with previous studies [37], which have shown that when lesions are located in the corpus callosum and cingulate gyrus, the impairment in information processing speed (SDMT) is more pronounced. We also found that WMLN is negatively correlated with verbal fluency (COWA). The COWA task requires rapid thinking and the generation of words related to a given letter or category. This process involves multiple cognitive functions, such as memory retrieval, language production, and verbal fluency. The higher the WMLN, the more likely it is that the neural network nodes within the brain are affected, leading to more significant cognitive impairment. In particular, verbal fluency tasks require the coordinated functioning of multiple brain regions. The more lesions there are, the more severe the impairment in these regions, resulting in decreased verbal fluency.

It is important to note that while the ALPS index is widely employed to assess the diffusion properties of perivascular spaces and serves as a surrogate for glymphatic function, the dynamics of the glymphatic system and cerebral waste clearance are extremely complex. Consequently, multiple assessment approaches have been developed to more comprehensively evaluate GS function [38]. Other techniques, such as choroid plexus volumetry [39] and perivascular space volume (PVS) [40] measurement, offer more holistic perspectives for evaluating glymphatic function. Additionally, global blood-oxygen-level-dependent signals and cerebrospinal fluid (gBOLD-CSF) imaging reveals cerebrospinal fluid dynamics by analyzing correlations between low-frequency BOLD signals and CSF movement [41].

There are several limitations in this study. First, the sample size of this study is relatively limited, which may affect statistical power. Moreover, since the study subjects were RRMS patients recruited from a single center, the generalizability of the results needs to be further validated in larger, multicenter cohorts. Second, this study adopted a cross-sectional design, which identified correlations between the ALPS index and multiple cognitive function indicators but could not infer causality. Future studies should employ longitudinal designs to explore the dynamic relationship between glymphatic function and cognitive changes over the course of the disease. Third, this study did not systematically incorporate variables such as patients' sleep status, fatigue level, and psychological state. Previous studies have suggested that these factors may also influence cognitive performance and brain clearance function. Future research should further include these potential confounding factors for control.

## Conclusion

This study demonstrates that the ALPS index is closely related to cognitive impairment in patients with relapsing–remitting multiple sclerosis (RRMS) and may serve as an independent discriminating factor of cognitive deficits. These findings provide new insights into the underlying pathological mechanisms and offer a basis for developing targeted interventions aimed at the glymphatic system.

## Data Availability

The data generated in this study is available from the corresponding author on reasonable request.
